# Use of Digital-Conventional Method for Managing a Patient with Severely Worn Dentition: A Clinical Report

**DOI:** 10.1155/2018/8456143

**Published:** 2018-11-19

**Authors:** Saied Nokar, Amirreza Hendi, Yasamin Babaee Hemmati, Mehran Falahchai

**Affiliations:** ^1^Associate Professor, Dental Research Center, Department of Prosthodontics, Faculty of Dentistry, Tehran University of Medical Sciences, Tehran, Iran; ^2^Assistant Professor, Dental Sciences Research Center, Department of Prosthodontics, School of Dentistry, Guilan University of Medical Sciences, Rasht, Iran; ^3^Assistant Professor, Dental Sciences Research Center, Department of Orthodontics, School of Dentistry, Guilan University of Medical Sciences, Rasht, Iran

## Abstract

Severe forms of attrition are frequently found in patients with no or insufficient posterior occlusal support. Management of such patients using fixed or removable prostheses is a complex procedure and is still a challenge for clinicians. The present clinical report describes step by step full mouth rehabilitation of a patient with severely worn dentition using computer-aided design/computer-aided manufacturing- (CAD/CAM-) generated wax patterns, milled zirconia frameworks, and fabrication of removable partial denture (RPD) abutments using a digital-conventional method. The results were satisfactory during 18 months of follow-up.

## 1. Introduction

Tooth wear is the process of losing the tooth structure due to noncarious lesions [1]. It is a normal phenomenon that progresses with aging [2]. However, multiple factors may need to be considered the reasons for excessive tooth wear and may include esophageal reflux, eating disorders, heavy masticatory forces, excessive consumption of acidic and raw foods, and altered pattern of chewing due to tooth loss or use of prosthesis [3].

Management of patients with worn dentition and loss of posterior tooth support by the use of fixed or removable prostheses is a complex procedure [4]. Articulated study casts and a diagnostic wax-up are the prerequisites for definitive diagnosis and treatment planning. Full mouth reconstruction while controlling for the causative factors would be a suitable treatment option [5]. Assessment of the vertical dimension is important for the management of such cases, and careful comprehensive treatment planning should be performed individually for each case [2].

Condyle dislocation and anterior movement of the mandible are related to the loss of posterior tooth support in class I partially edentulous patients. The lower incisal edges will be driven forward, and excessive forces will be applied to the palatal surface of the anterior maxillary teeth, thereby causing palatal surface loss. This phenomenon will be continued until the pulpal exposure occurs. Therefore, it should be mentioned as an important force factor in partially edentulous patients. Thus, establishing the posterior occlusal support should be considered a prerequisite for any prosthetic treatment in such patients [6].

There are several options that can be used for managing tooth wear patients including Dahl concept, direct composite restorations, and indirect restorations. However, there are no definite material and technique for such patients supported by evidence [7, 8]. Removable partial dentures (RPDs) and surveyed crowns have been used as definitive treatments for patients without posterior support for many years [9]. Systematic reviews have concluded that using RPD in arches with anterior and premolar teeth generally fulfills the requirements of a functional dentition, especially in older patients [10, 11]. Surveying the restoration to ensure the presence of the planned contour is a mandatory procedure in the laboratory process [12]. The rapid progress in computer-aided design/computer-aided manufacturing (CAD/CAM) technology has led to a huge impact on all fields of dentistry particularly prosthodontics [13]. Survival rates of CAD/CAM-generated tooth and implant-supported prostheses appear to be similar to those of conventionally fabricated dentures [14]. Metal-ceramic restorations usually have been used as surveyed crowns in prosthetic rehabilitation. The retentive portion of the labial or buccal surfaces in metal-ceramic restorations is made of the veneering porcelain, and the functional areas (guiding plane and rest seat) are made of metal [15]. Fabrication of the wax pattern is a critical step in this procedure [16]. It is now possible to fabricate the shape and contour of the wax pattern with different computer-aided systems such as subtractive or additive systems, which do not have many of the limitations of the conventional waxing technique [16, 17]. Various designs such as copings, full anatomical crowns, and fixed partial dentures (FPDs) can be applied using different software programs from different manufacturers [13].

Several reports are available on prosthetic rehabilitation of a patient with severely worn dentition. However, considering the advances in dental materials and techniques for the fabrication of restorations, there is still a need for more studies using newer methods. Herein, we report step by step prosthetic rehabilitation of a patient with worn dentition.

## 2. Case Presentation

**Figure 1:**
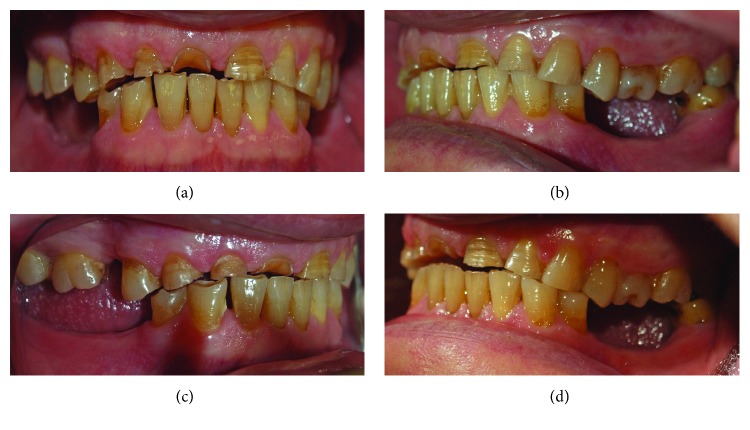
Preoperative images: (a) fontal view; (b, c) lateral views; and (d) opposing tooth facets in eccentric movement.

**Figure 2:**
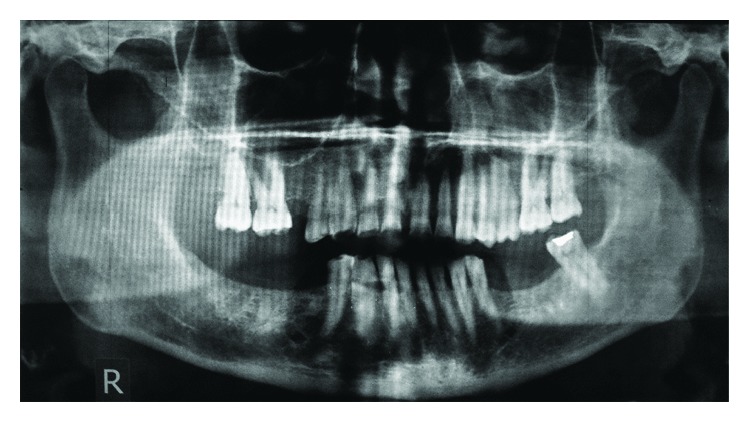
Preoperative radiograph.

A 57-year-old woman was referred to the Prosthodontics Department of the School of Dentistry, Tehran University of Medical Sciences, for treatment of her worn dentition. The chief complaint of the patient was speech difficulties and reduced chewing ability. The patient had a history of osteoporosis and also diabetes mellitus, which were under control. After patient interview, it was found that she was taking oral bisphosphonates over the past year. The left second molar had been restored with a class I amalgam restoration. In extraoral examination, the patient's face was symmetrical, the facial proportions were equal, and the lips were competent. There were no signs or symptoms of temporomandibular joint disorder. In intraoral examination, severe wear was found in maxillary anterior teeth, and other teeth had mild occlusal wear (Figures 1(a)–1(c)). In eccentric movements, the opposing tooth facets were matched confirming the presence of attrition (Figure 1(d)). In radiographic examination, a remaining root related to the left mandibular second molar was found. The root had been surrounded completely by the intact bone with no clinical or radiographic signs/symptoms. After consulting with an oral and maxillofacial surgeon and since the patient was taking bisphosphonates, it was decided to avoid any aggressive intervention and the root remained untouched. No other remarkable findings were observed (Figure 2).

For vertical dimension (VD) analysis, facial appearance, interocclusal rest, and phonetics were evaluated. The vertical dimension of rest (VDR) was approximately 6 mm greater than the VDO, which was greater than the normal range (2–4 mm), and the closest speaking space was about 3 mm. Therefore, it was possible to gain restorative space by increasing it. Periodontal examination of the teeth was performed. Before any procedure, dental prophylaxis was performed, and the patient received oral hygiene instructions.

**Figure 3:**
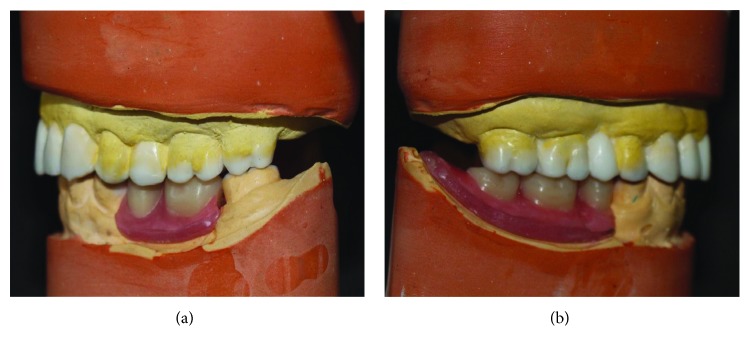
(a, b) Diagnostic wax up in lateral views.

Alginate impressions (Tropicalgin, Zhermack, Badia Polesine, Rovigo, Italy) were made from both arches to obtain the diagnostic casts. The base record with a wax rim was fabricated for the mandibular cast. The vertical dimension of occlusion (VDO) was established using the described methods. The required amount of VDO increase was approximately 2 mm. An interocclusal record was made with zinc oxide eugenol paste (Luralite, Kerr Corp., Orange, CA, USA) on wax rims using an anterior deprogramming device in the considered VDO. A face bow record was used to mount the maxillary cast in relation with the transverse condylar axis. In the next step, the mandibular cast was mounted against the opposing arch on a semiadjustable articulator (Denar Mark II, Whip Mix Corporation, Louisville KY, USA) by centric relation bite record. The check bite records were obtained to set the condylar elements. First, the mandibular anterior wax-up was performed. After the intraoral verification of the mandibular canine wax-up with the corner of the mouth and relationship of the lip to the teeth, a Broadrick occlusal plane analyzer was used to determine the occlusal plane. Then, the wax-up procedure and mandibular posterior tooth setup were accomplished and the mandibular interim removable partial denture was fabricated (Figure 3). Duplicate casts were obtained from the diagnostic wax-up, and mockup shells were fabricated (Drufolen H; Dreve Dentamid GmbH, Unna, Germany). Shells were used to fabricate directly bonded build-ups for lower anterior and all of the upper teeth, and the mandibular interim removable partial denture was delivered. The occlusal centric stops and the anterior guidance were adjusted. Also, the smile line and phonetics were verified. For three months, the patient's condition was assessed in terms of the temporomandibular joint and muscle comfort, phonetics, chewing ability, and esthetics. Next, the available treatment options were discussed with the patient. Since the patient used oral bisphosphonates for more than 3 months in the past year and according to the consultation with her attending physician, implant treatment and surgical procedures were excluded. According to the diagnostic procedure, full-coverage restorations were chosen for the upper jaw. A RPD was chosen for the lower jaw to restore the posterior segment. Porcelain veneer laminates and full coverage restorations were considered for the mandibular anterior teeth and the main abutment of the RPD, respectively. Because of the lack of restorative space, full metal restoration was chosen for the left second molar. Since an appropriate posterior support was essential for such a patient to prevent the wear induced by protrusive interferences, it was decided to restore the mandibular arch first, with a definitive RPD to ensure posterior support.

### 2.1. Rehabilitation of Mandibular Arch

**Figure 4:**
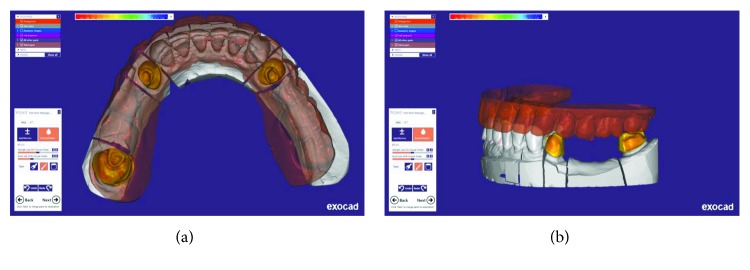
Mandibular restoration digital design: (a) occlusal view and (b) lateral view.

**Figure 5:**
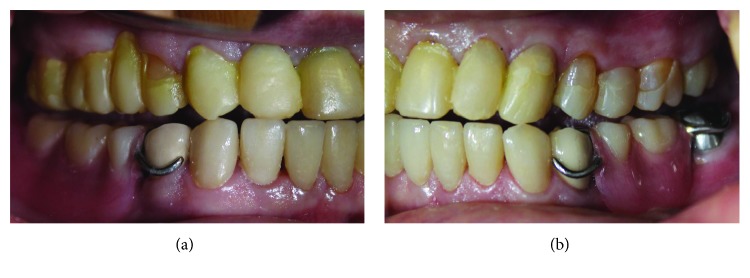
Establishment of mutually protected occlusion after mandibular restoration delivery: (a) right side and (b) left side.

Tooth preparation was performed using a putty index made from the diagnostic wax-up. Anterior lower teeth were prepared for the porcelain laminates with light chamfer finishing line, and the other teeth were prepared for metal ceramic restorations with a deep chamfer margin to serve as RPD abutments. A complete arch putty-wash impression was made with polyvinylsiloxane (Panasil, Kettenbach, Hesse, Germany) after retracting the gingiva using the retraction cords. The temporary restorations were made and cemented using temporary cement (Temp Bond, Kerr Corp., Orange, CA, USA). The impression was poured, and the base record and wax rim were fabricated on the definitive cast. In the next appointment, an impression was made from the mandibular temporary restorations to be used as an index for the fabrication of final restorations. After removal of temporary restorations, interocclusal record was made using an anterior deprogramming device and zinc oxide eugenol paste on wax rims in centric relation. A2 shade heat-pressed lithium disilicate glass-ceramic (IPS e.max press, Ivoclar Vivadent, Liechtenstein) was chosen for the fabrication of the laminates. In laboratory procedures, the definitive cast and the cast from temporary restorations were scanned by a 3Shape laboratory scanner (D700; 3Shape A/S; Copenhagen, Denmark). It was decided to use CAD/CAM-generated wax patterns. Therefore, digital designing software (Exocad DentalCAD, Exocad, Darmstadt, Germany) was used to prepare the wax patterns according to the cast from finalized temporary restorations. For RPD abutments, it was tried to survey the guide planes digitally as well (Figure 4). Wax patterns were milled using a milling machine (Arum milling machine, Dowoom, Daejeon, Korea). Wax patterns of RPD abutments were surveyed and refined conventionally when necessary. The metal frameworks of RPD abutments were tried in after resurveying. Finally, porcelain veneering was done and final restorations were cemented. A mutually protected occlusion was established for the patient. In the next step, the procedures for RPD fabrication were started. The primary cast was obtained from the preliminary impression. Definitive impression was made with polyvinylsiloxane (Monopren, Kettenbach, Hesse, Germany) using a custom tray. In RPD framework try-in appointment, the altered cast technique was applied by making an impression from the free-end region with an acrylic tray attached to the framework. Finally, after tooth arrangement try-in, the definitive RPD was processed and delivered to the patient. A mutually protected occlusion was established (Figure 5).

### 2.2. Rehabilitation of Maxillary Arch

**Figure 6:**
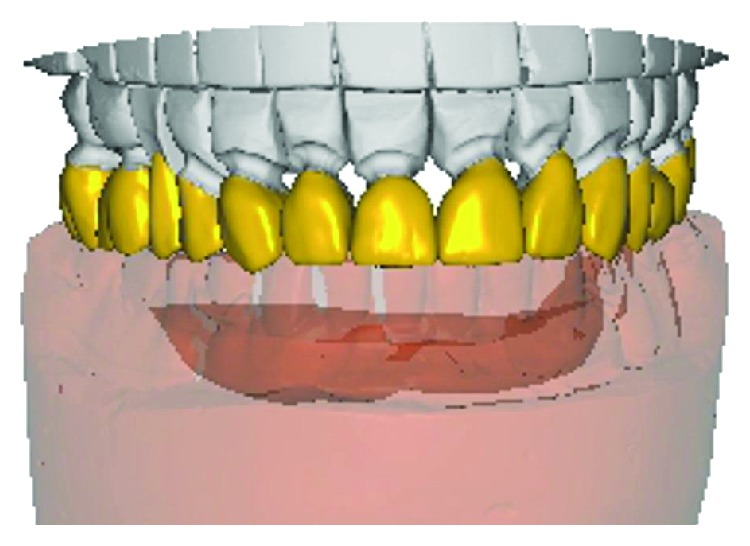
Full contour design of maxillary restorations.

**Figure 7:**
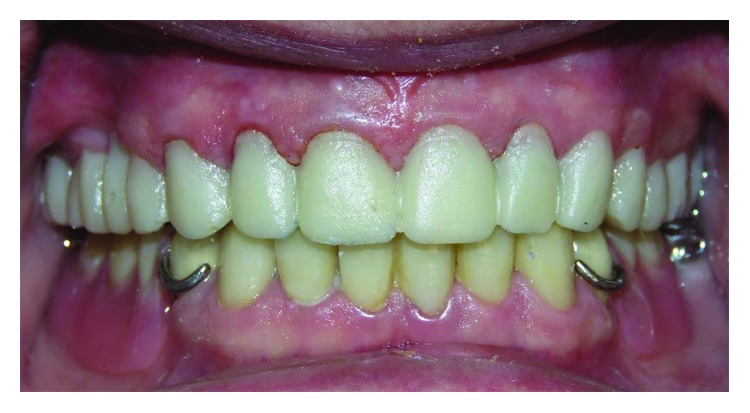
Maxillary temporary restorations.

**Figure 8:**
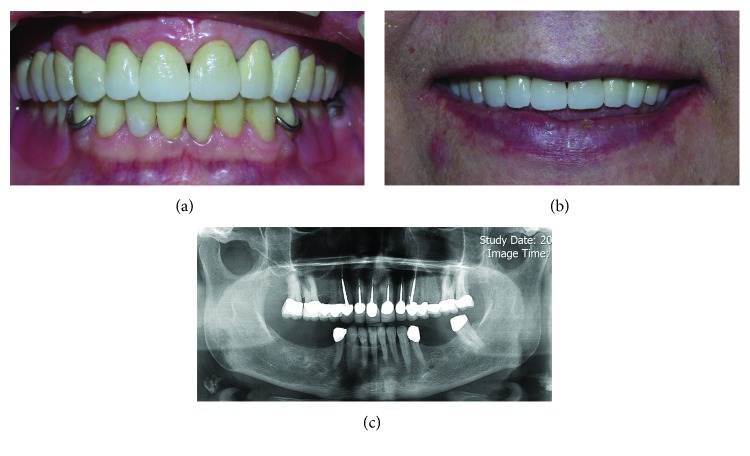
Postoperative images: (a) intraoral view, (b) smile view, and (c) postoperative panoramic view.

Root canal therapy was performed for the maxillary anterior teeth. Then, casting post and cores were made according to the wax-up index and the opposing arch and cemented with Panavia F2 (Kuraray Noritake Dental Inc.). Tooth preparation was done with a round shoulder margin and checked with the index from diagnostic wax-up. Temporary restorations were made using mockup shell and cemented with zinc oxide temporary cement (Temp Bond, Kerr Corp., Orange, CA, USA). Then, mutually protected occlusion was established. In the next appointment, the temporary restorations were finalized by checking the occlusal contacts, phonetics, and esthetics, and an impression was made from the temporary restorations (Figure 6). The final impressions were made with a 2-step impression technique (putty and light body impression materials) (Panasil, Kettenbach, Hesse, Germany) after gingival retraction. Definitive cast was poured, and an interocclusal record was obtained to mount the cast on a semiadjustable articulator. A laboratory scanning procedure was accomplished, and A2 shade zirconia frameworks (IPS e.max ZirCAD, Ivoclar Vivadent, Liechtenstein) were designed according to the temporary restorations verified in the clinic (Figure 7). Try-in of the frameworks was performed, and then porcelain veneering was done using customized anterior guide table. After porcelain try-in appointment and establishing mutually protected occlusion, the restorations were stained and glazed. The final restorations were cemented with glass ionomer cements (GC Fuji II, GC America, Illinois, USA) (Figure 8). Oral hygiene instructions were given to the patient, and follow-up appointments were scheduled. No problem was reported by the patient during 18 months of follow-up.

## 3. Discussion

Randomized clinical trials on prosthetic rehabilitation of patients with worn dentition are rare. Lack of evidence about the long-term clinical outcome of treatment may complicate the process of clinical decision-making [1]. Implant prostheses or RPDs are the treatment of choice for patients who have lost posterior occlusal support [6]. Prosthetic rehabilitation of patients who have lost anterior guidance and posterior occlusal support using anterior restoration and posterior RPD has been recommended for those with financial problems or special medical needs. However, the restored anterior teeth can be easily exposed to excessive occlusal loads if the patient refuses to wear the RPD [2].

The use of a ceramic veneer is common for reestablishing the leading edges of the lower anterior teeth when the labioincisal angle has been worn out. Wear resistance is an important factor for restoration longevity. It may directly affect the long-term maintenance of stable occlusal contacts. According to the study by Zhi et al., [18], the wear resistance of composite resin blocks was significantly lower than that of a ceramic block in contact with the enamel. Therefore, composite resin restorations cannot be used in mandibular anterior teeth of our patient due to lack of wear resistance.

The application of CAD/CAM technology may be useful to improve the adaptation of the axial walls and decrease the wax pattern marginal gap [12]. According to Shamseddine et al., [16], the subtractive CAD-CAM waxing technique resulted in an improved fit of a pressed lithium disilicate crown compared to the conventional waxing technique. In this report, fabrication of the wax pattern for ceramic veneers (for pressed lithium disilicate) and surveyed crowns (for MCR) was done by the CAD/CAM technology. However, due to the lack of a special software design for surveyed crowns, the milled patterns had some deficiencies especially in terms of parallelism of guide plans. Therefore, a digital-conventional method was used to fabricate the surveyed crowns.

Stabilized zirconia was used for maxillary full ceramic crowns due to its optimal properties, high survival rate, and esthetic considerations [5]. When marginal and internal fits were compared between the CAD/CAM and slip-cast zirconia frameworks, higher axial, occlusal, and marginal adaptations were seen in the CAD/CAM group [6]. Therefore, a milling technique was used to make definitive restorations for the patient's upper teeth.

Educating the patient on wearing RPD is an important factor in cases that have lost posterior stop, as it has been shown that patient compliance to wear distal-extension base RPDs is poor. Regular follow-up appointments for occlusal adjustment and checking the fit of RPD are the most important issues in the long-term success of this treatment. Any sign of recurrent wear should be identified and controlled as soon as possible [1, 2].

## 4. Conclusion

Full mouth rehabilitation of a patient especially with severely worn dentition is a complicated procedure. Considering the recent developments in all fields of dentistry, the clinicians have many options in terms of the materials and methods which make it necessary to use an accurate step-by-step approach. In our patient, various methods and materials were used to obtain the best result. One of the new and innovative methods was using digital designing for the fabrication of the wax pattern of RPD abutments. Based on our experience in this case, it is not possible to obtain the perfect RPD abutment restoration using only the digital processing technique, and there is a need for specific software to design and survey the RPD abutments.
